# Long non‐coding RNAs in the spinal cord injury: Novel spotlight

**DOI:** 10.1111/jcmm.14422

**Published:** 2019-05-29

**Authors:** Zheng Li, Idy H. T. Ho, Xingye Li, Derong Xu, William K. K. Wu, Matthew T. V. Chan, Shugang Li, Xiaodong Liu

**Affiliations:** ^1^ Department of Orthopaedic Surgery Peking Union Medical College Hospital, Chinese Academy of Medical Sciences and Peking Union Medical College Beijing China; ^2^ Department of Anaesthesia and Intensive Care, Faculty of Medicine The Chinese University of Hong Kong Hong Kong Hong Kong; ^3^ Department of Orthopedic Surgery Beijing Jishuitan Hospital, Fourth Clinical College of Peking University, Jishuitan Orthopaedic College of Tsinghua University Beijing China; ^4^ Department of Orthopedics The Affiliated Hospital of Qingdao University Qingdao China; ^5^ State Key Laboratory of Digestive Diseases, Faculty of Medicine Institute of Digestive Diseases and LKS Institute of Health Sciences, The Chinese University of Hong Kong Hong Kong Hong Kong

**Keywords:** glial activation, lncRNA, neuronal death, spinal cord injury, transcriptome

## Abstract

Spinal cord injury (SCI) may lead to persistent locomotor dysfunction and somatosensory disorders, which adversely affect the quality of life of patients and cause a significant economic burden to the society. The efficacies of current therapeutic interventions are still far from satisfaction as the secondary damages resulting from the complex and progressive molecular alterations after SCI are not properly addressed. Recent studies revealed that long non‐coding RNAs (lncRNAs) are abundant in the brain and might play critical roles in several nervous system disorders. At the cellular level, lncRNAs have been shown to regulate the expression of protein‐coding RNAs and hence participate in neuronal death, demyelination and glia activation. Notably, SCI is characterized by these biological processes, suggesting that lncRNAs could be novel modulators in the pathogenesis of SCI. This review describes recent progresses in the lncRNA transcriptome analyses and their molecular functions in regulating SCI progression.

## 1 INTRODUCTION

Spinal cord injury (SCI) is one of the most devastating neurological diseases affecting between 250 000 and 500 000 people worldwide annually (http://www.who.int/en/news-room/fact-sheets/detail/spinal-cord-injury). Primary SCI is commonly caused by direct trauma (eg contusion and compression during vehicle incidents, falls, violence or sports) and pathological alterations (eg cancers),^1^ resulting in immediate haemorrhage and rapid neuronal cell death. This is followed by secondary injury mechanisms, including glutaminergic excitotoxicity, oxidative stress, increased adaptive immune responses, Wallerian degeneration and scar tissue formation, leading to further structural and functional disturbances that spread spatially from the site of initial injury.^2^, ^3^, ^4^ These biochemical alternations can be further divided into acute, subchronic and chronic phases, which require tailored therapeutic strategies.^5^ An essential problem is that adult spinal cord has a low regenerative capacity. This results in paralysis or movement dysfunctions, sensation deficits and autonomic dysfunctions, such as loss of urinary and bowel functions. Unfortunately, current treatments are insufficient due to multiple and complex aetiologies of SCI. Further understandings of cellular and molecular mechanisms of primary and secondary injuries are necessary for finding a new therapeutic strategy to promote functional recovery of patients with SCI. In this regard, long non‐coding RNAs may provide hints for novel treatment strategies for SCI.

**Figure 1:**
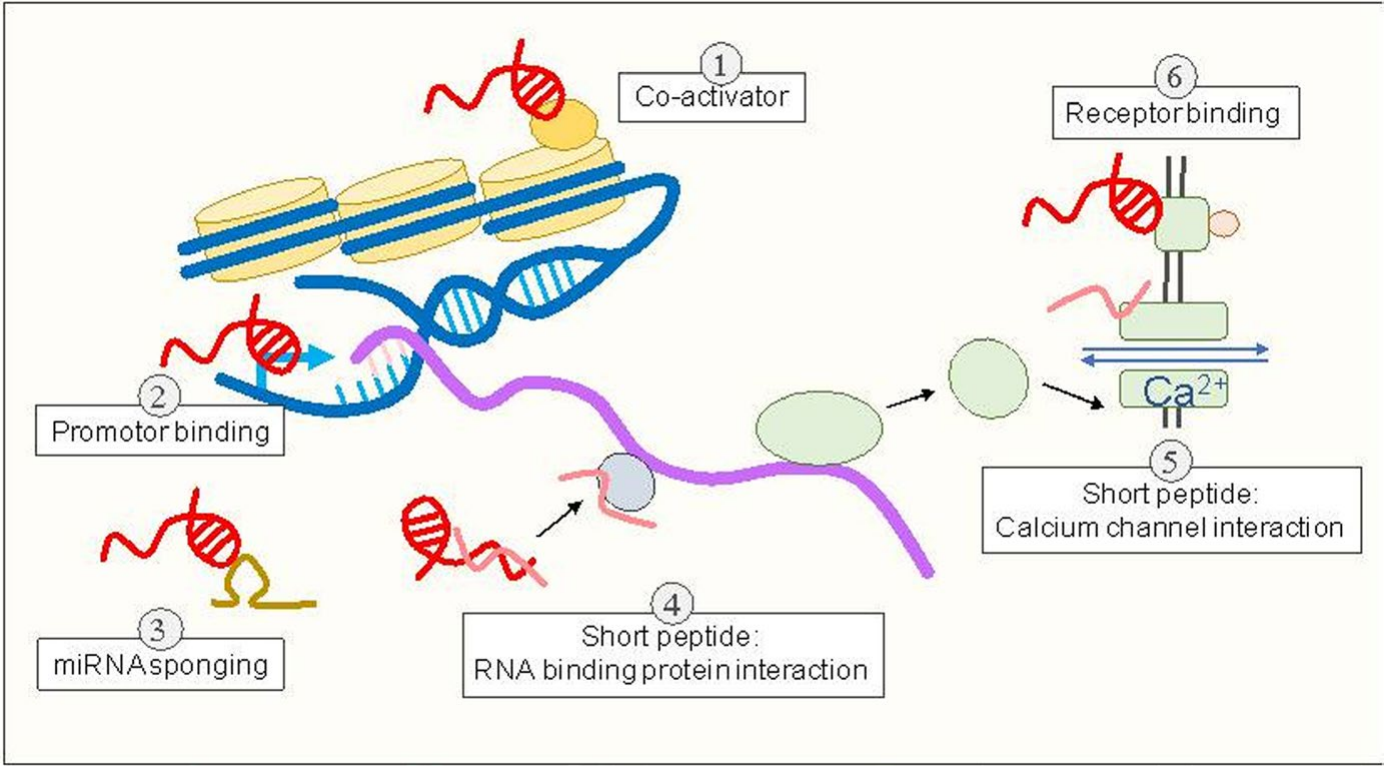
Summary of lncRNA functions at different regulatory levels. (1) lncRNA transcripts recruit chromatin modifiers as a co‐activator to regulate subsequent chromatin compaction for gene silencing. (2) Transcriptionally, lncRNA leads to either gene repression or activation by hybridization to promoter regions of gene loci. (3) Post‐transcriptionally, lncRNAs act as ceRNAs to regulate RNA transcripts by miRNAs sponging. (4) Short peptides encoded by lncRNAs interact with RNA binding proteins to modulate protein function. (5) Alternatively, short peptides interact with calcium channels for protein function modulation at the Post‐translational level. (6) lncRNAs bind directly to receptors to regulate its ion channel activity

Long non‐coding RNAs (lncRNAs) were identified as non‐protein‐coding transcripts that consist of more than 200 nucleotides.^6^ lncRNAs were initially considered as transcriptional noise that was transcribed by the RNA polymerase II complex.^7^ However, recent studies demonstrated that lncRNA retained limited protein‐coding capacity to encode short peptides.^8^ LNCipedia (https://lncipedia.org/) is a public and active database that aims to record and annotate lncRNA sequences and structures. Since its establishment in 2013,^9^ five updated databases have been published.^10^ Currently, a total of 127 802 long non‐coding transcripts and 56 946 lncRNAs are curated by LNCipedia. Accumulating evidences revealed that many lncRNAs may functionally interact with proteins, adding a new dimension to the physiological and pathological roles of genes coding. lncRNAs play multiple roles in gene expression (Figure 1). Firstly, lncRNAs may locally (in cis) or non‐locally (in trans) modulate gene transcription. Polycomb Repressive Complex 2 (PRC2) is required for the initiation of histone modifications and subsequent chromatin compaction.^11^ By interacting with PRC2, lncRNA transcripts, such as X‐inactive specific transcript (XIST) and HOX Antisense Intergenic RNA (HOTAIR), were shown to regulate chromatin structure and silence gene transcription.^12^, ^13^, ^14^ Meanwhile, lncRNAs may complementarily hybridize to promoter regions of gene loci, leading to either repression or activation of gene transcription. An example of this scenario is the transcription of *PDCD4* whose promoter interacts with lncRNA CASC9, thereby recruiting the transcription repressor EZH2.^15^ For its co‐activator role, lncRNA Evf‐2 promoted the recruitment of chromatin‐binding protein Dlx‐2 and then enhanced the transcription of Dlx‐6 gene.^16^ Secondly, lncRNAs may regulate gene expression at the post‐transcriptional level. For instance, lncRNA can directly bind to coding RNA transcripts and modulate translation, degradation, splicing and editing of targets.^17^, ^18^ Alternatively, short peptides encoded by lncRNAs were recently demonstrated to interact with RNA binding proteins or calcium channels and hence modulate protein functions.^19^, ^20^ Moreover, it has been reviewed that lncRNAs may act as competing endogenous RNAs (ceRNAs) to regulate coding RNA transcripts by sponging microRNAs.^21^ Last but not least, lncRNA‐mediated post‐translational regulations were drawing attention. Evidence have shown that lncRNA might directly bind to purinergic receptor P2X7 and potentially regulate its ion channel activity.^22^

Given the enormous amounts of lncRNAs and their profound effects on coding RNAs, it is notsurprising that lncRNAs may play important roles in pathogenesis. It is worthwhile to note that up to 40% of all the known lncRNAs are specifically expressed in the brain,or possibly other parts of the central nervous system.^23^ This finding indicated that lncRNAs, especially differentially expressed lncRNAs, might provide additional regulations in the pathogenesis of nervous system diseases. In this regard, identifications of differentially expressed lncRNAs using genome‐wide approaches were a good start to understand lncRNA‐ mediated disease development. In this review, differentially expressed lncRNAs profiling in murine models of SCI will be summarized. Emerging evidence of the interplay between lncRNA function and SCI will also be highlighted.

## 2 LNCRNA EXPRESSION PROFILING IN SCI

**Figure 2:**
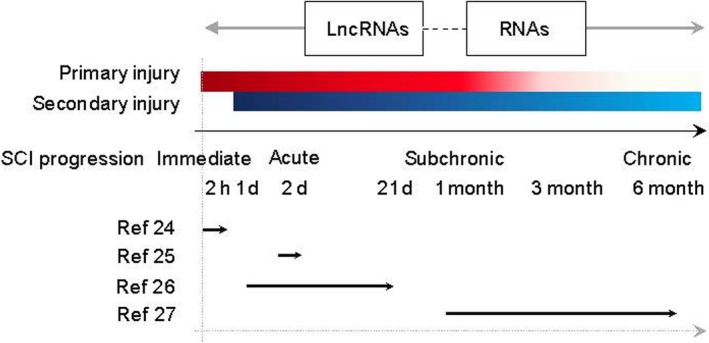
Involvement of lncRNAs in the progression of spinal cord injury. lncRNAs are highly dynamic, spatially and temporally in acute, subchronic and chronic phases of spinal cord injury (SCI), shown by four transcriptome analyses focusing on lncRNA dysregulation after SCI. Stage‐specific LncRNA‐mRNA co‐expression networks were involved in and associated with pathological changes during SCI progression

Secondary injury‐related neuropathological changes are gradually developed after primary SCI. The choices of regimens and clinical outcomes highly rely on the status of SCI. Gene profiling in different phases of SCI is necessary and may provide insights into its dynamic gene network and identify phase‐specific druggable targets during the progression of the disease. To date, only four transcriptome analyses have focused on lncRNA dysregulation after SCI. All of these studies have introduced a contusion to thoracic spinal cord and the epicentre injuries were collected for gene profiling. Due to the different platforms (microarray vs RNA sequencing) and different species (mouse vs rat) used, it is difficult to compare the lncRNA profiles among four studies (Table 1). Surprisingly, two studies, which were conducted by the same team, presented no overlap between the top 20 differentially expressed lncRNAs in immediate (2 hours)^24^ and acute (2 days)^25^ SCI stages. These results suggested that lncRNA expression was highly dynamic across different stages. Nonetheless, these studies have aimed to reveal the lncRNA profiles in different post‐injury time‐points, from immediate,^24^ acute,^25^, ^26^ subchronic^27^ to chronic^27^ phases after SCI (Figure 2). These studies provided preliminary views on stage‐specific lncRNA modulation.

**Table 1:** Long non‐coding RNA expression profiles in spinal cord injury

Ref	Method	Species	SCI model	Site of samples	Post‐SCI phases	Sample collection^a^	Threshold	Up‐regulated^b^	Down‐regulated^c^
24	Microarray	Rat	T10 contusion	Injury epicentre	Immediate	2 h	FC ≥ 2 *P* ≤ 0.05	528	244
25	Microarray	Rat	T10 contusion	Injury epicentre	Acute	2 d	FC ≥ 2 *P* ≤ 0.05	1332	1861
26	Microarray	Mouse	T10 contusion	Injury epicentre	Acute	1 d 3 d 7 d 21 d	FC ≥ 2 FDR ≤ 0.05	164 212 326 141	181 290 565 40
27	RNA Seq	Rat	T9 contusion	Injury epicentre	Subchronic Chronic	1 mo 3 mo 6 mo	FC > 2 FDR < 0.01	120 162 125	17 77 54

Abbreviations: FC, fold change; FDR, false discovery rate.

a Post‐injury time for sample collection.

b Number of up‐regulated lncRNAs.

c Number of down‐regulated lncRNAs.

The first study focusing on lncRNAs was performed to identify differentially expressed lncRNAs in a period of 1‐21 days (acute to subchronic phase) after a moderate contusion SCI^26^. Hundreds of lncRNAs were up‐regulated or down‐regulated at all time‐points (ie 1, 3, 7 and 21 days) after injury. This suggested that lncRNAs expression was sensitive to the pathological changes across acute and subchronic phases of SCI. It is not surprising to find that some of these lncRNAs might participate in the pathological changes. Similar to coding RNAs, the number of differentially expressed lncRNAs at the epicentre of injury gradually increased on day 1 and day 3, peaked on day 7 and then recovered on day 21. Interestingly, co‐expression analysis using gene quantification values of different time‐points demonstrated that many lncRNAs expression levels were highly correlated with differentially expressed coding RNAs (coefficients of correlation > 0.997). The lncRNA‐mRNA co‐expression network was then constructed with these coefficients of correlation. This further revealed that several lncRNAs had high degrees and K‐core values and belong to the ‘hub’ nodes of co‐expression network. In graph theory, higher degrees and K‐core values indicated that a node (gene) is connecting with higher number of other nodes in the network or subnetwork (K‐core).^28^.The network analysis highlighted that these hub lncRNAs correlated with a substantial amount of coding genes, in terms of expression levels. Further experiments, such as effects of overexpressing or knockdown of these hub lncRNAs on SCI‐associated transcriptome in relation to histological and functional recovery, will be required. Neither were functional enrichments of co‐expressed coding mRNAs annotated. The functional insights of differentially expressed lncRNAs in the progression of SCI were lacking.

Recently, a systematic analysis focusing on differentially expressed lncRNAs in subchronic (1‐3 months) and chronic (6 months) phases of moderate (150‐kdyn) contusive SCI in rats was done.^27^ Compared to sham control, a total of 277 lncRNAs were identified to be differentially expressed at all the time‐points of the study, ie 1, 3 and 6 months after SCI. A co‐expression network was then constructed using the 277 lncRNAs and the mRNAs that were significantly correlated. Gene Set Enrichment Analysis (GSEA) revealed that these highly correlated protein‐coding RNAs were enriched in various functionalities, including signalling transduction, immune response, epigenetic modification, nervous system and extracellular matrix. The enrichment is tightly associated with specific process of SCI pathogenesis. For instance, the protein‐coding RNAs in the lncRNA‐mRNA network were enriched for ‘myelin sheath,’ ‘astrocyte development’ and ‘gliogenesis.’ Apparently, this is parallel to chronic demyelination and scar consolidation that is observed in chronic SCI.^3^, ^29^, ^30^ As discussed above, these highly correlated protein‐coding mRNA could be targets of differentially expressed lncRNAs. Functional annotations of these mRNA hence provide an insight into their potential actions in subchronic and chronic SCI. Another and maybe a superior approach for the functional annotations of differentially expressed lncRNAs is to identify the protein‐coding genes that are spatially close to these lncRNA as lncRNAs are frequently reported to cis‐regulate the transcription of coding neighbours.^14^ Based on this theory, the lncRNAs with potential modulatory roles were chosen with two criteria: (a) coding neighbours (usually within 5 kb in the genome) are differentially expressed between control and SCI; and (b) lncRNAs and coding RNAs are significantly correlated (Pearson's correlation, *P* < 0.05) at the expression level. This analysis generated a list of 77 lncRNAs with high chance of functionality in chronic SCI. For example, LOC102547088 was identified as an up‐regulated lncRNA to potentially promote the expression of pro‐apoptotic gene *Tchp* in chronic SCI. These in silico analyses shed new lights on the mechanism of lncRNA‐mediated SCI modulations and future direction of functional studies. Finally, another intriguing and clinically relevant analysis investigated the connection between differentially expressed lncRNA and disease‐associated single nucleotide polymorphisms (SNPs). A large number of genetic association studies have identified massive SNPs that are associated with the risk of diseases or symptoms.^31^, ^32^, ^33^ The majority of these SNPs is distributed in intergenic and intronic regions of the genome. It is therefore difficult to interpret the effects of disease‐associated SNPs in terms of molecular function. Recent advances in lncRNAs, however, suggested a novel approach for functional studies of these SNPs. It is now clear that more than 80% lncRNAs are distributed in the SNP‐rich regions, ie the intergenic and intronic regions.^23^, ^34^ It is likely that alternative genotypes in SNPs may influence the molecular functions of lncRNAs and hence contribute to disease risk. In thechronic SCI model, 76% of the differentially expressed lncRNAs were mapped to the intergenic and intronic regions. Notably, 23 lncRNAs were homologous to human genomic regions containing SNPs that had been associated with neurodegenerative diseases. It is intriguing to investigate the effects of SNP‐based genotypes on lncRNA function. Alternatively, SCI‐associated SNPs may also lead to the identification of SCI‐related lncRNA‐mRNA networks for developing therapeutic regimens.

Although lncRNA profiling analysis may greatly accelerate the research of lncRNA‐mediated mechanisms underlying SCI, such studies are just starting from scratch. Currently, only a few studies focus on lncRNAs in animal models of SCI. Further studies investigating temporal (different post‐injury phases) and spatial (ie lesions, peri‐lesion area) changes, as well as comparing different models of SCI would certainly strengthen our understanding of SCI. Meanwhile, it should be noted that a large number of former transcriptome studies have been conducted on rat or mouse models of SCI. More than 700 of such studies are recorded in the NCBI GEO database (www.ncbi.nlm.nih.gov/gds). Although these previous studies were not concentrated in lncRNAs, many of them did contain expression data of lncRNAs. Retrieving these datasets is an efficient way to study lncRNAs. On the other hand, owing to the limited knowledge of lncRNAs, it is not easy to functionally annotate lncRNAs. The study performed by Duran and his college represented a good example of systematic analysis.^27^ LncRNA‐mRNA network reconstructions based on co‐expression coefficients or the identification of coding neighbours appear to be two essential analyses for subsequent pathways (eg KEGG or GSEA) and gene ontology annotations. Alternatively, weighted correlation network analysis (WGCNA) would be a good option.^35^ In addition to gene modules (clusters) that contain highly correlated lncRNAs and coding RNAs, WGCNA may determine particular phenotypes (eg time‐points of injury or gliogenesis) that are associated with these gene modules. Weighted correlation network analysis therefore may provide more clues from bioinformatic annotations. In this connection, cell‐type specific transcriptome analysis will be of great interest to researchers. By utilizing fluorescence‐activated cell sorting (FACS) technique and reporter mice lines, which conditionally express GFP in certain cell types, it is feasible to purify particular cells of interest for gene profiling.^36^, ^37^ With the advancement of single‐cell sequencing, it is also possible to investigate the potential involvement of lncRNAs in the functions of heterogeneous cell population after SCI. Nonetheless, experiments to disclose molecular functions of lncRNAs are absolutely neccessary for accurate systematic analysis on lncRNAs‐centered transcriptome.

## 3 FUNCTIONAL ROLES OF LNCRNAS IN SCI

**Figure 3:**
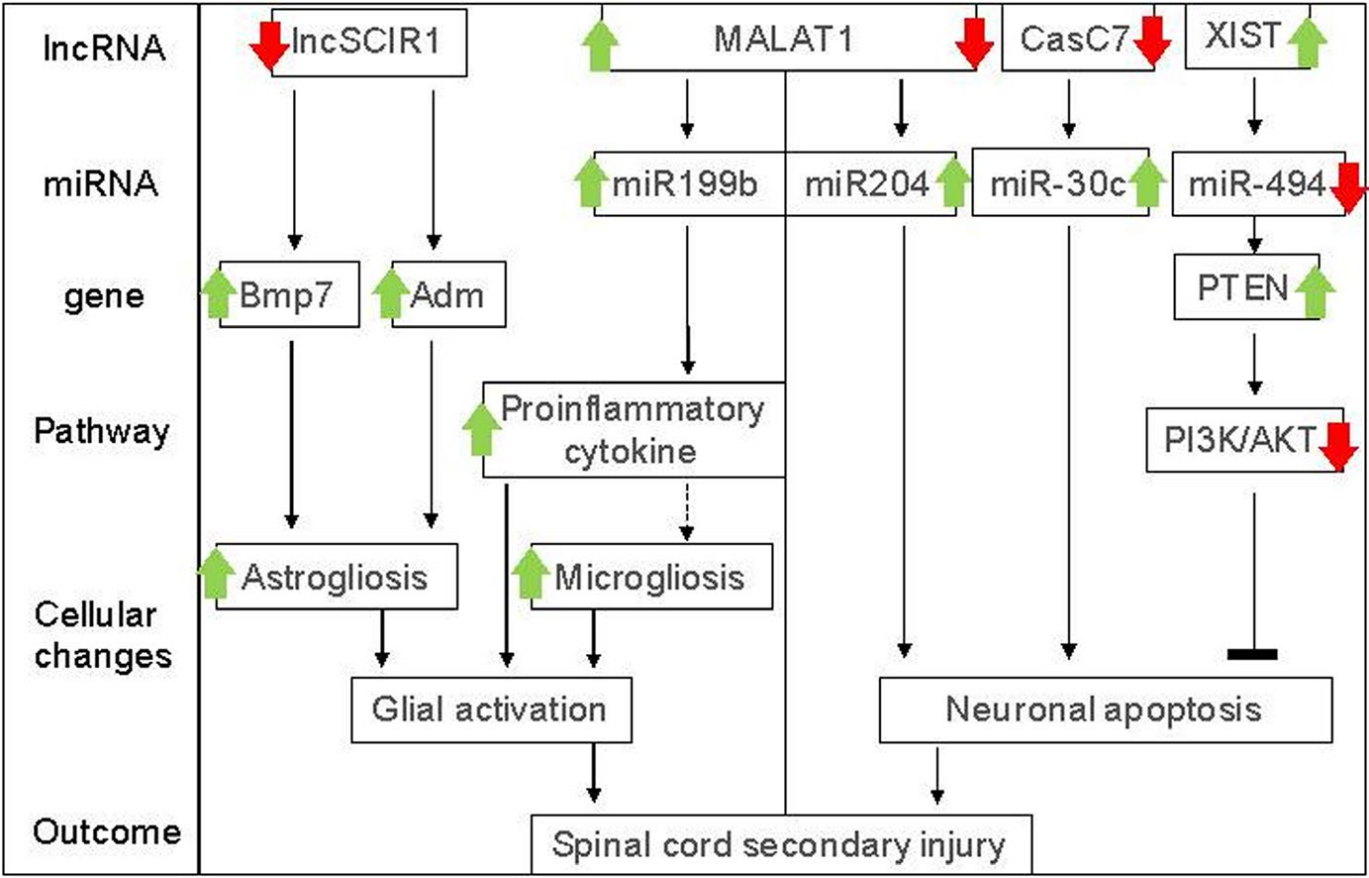
Known functional roles of lncRNAs on glial activation and neuronal apoptosis. Green box: Glial activation induced by lncRNAs in spinal cord injury (SCI). Down‐regulation of lncSCR1 in acute contusive SCI model led to up‐regulation of Bmp7 and Adm, resulting in astrogliosis. Up‐regulation of metastasis associated lung adenocarcinoma transcript 1 (MALAT1) in the same model sponged miR199b, leading to pro‐inflammatory cytokine production and microgliosis. Blue Box: Neuronal apoptosis regulated by lncRNAs in SCI. In the spinal cord ischaemic/reperfusion injury (SCIRI) model, suppression of MALAT1 sponged miR‐204‐dependent apoptosis, whereas that of CasC7 sponged miR‐30c‐dependent apoptosis. In the contusive SCI model, X‐inactive specific transcript (XIST) was up‐regulated, followed by miR‐494 down‐regulation and phosphatase and tensin homolog deletion on chromosome 10 (PTEN) activation‐induced PI3K/AKT pathway, resulting in neuronal protection against apoptosis

Recently, the biofunctional characterizations of differentially expressed lncRNAs in SCI were increasing. In particular, the modulation of glial activation and neuronal apoptosis by lncRNAs have become areas of intense investigation (Table 2; Figure 3).

**Table 2:** Functional characterization of the lncRNAs in spinal cord injury

lncRNAs	Regulation	Functional roles	Effectors	Reference
MALAT1	Up	Promotes pro‐inflammatory cytokines production in microglia	miR‐199b	44
lncSCIR1	Down	Inhibits migration and proliferation of astrocytes	Bmp7 Adm	45
XIST	Up	Neuronal death	miR‐494 PTEN PI3K/AKT	48
CasC7	Down	Neuroprotection	miRNA‐30c	49
MALAT1	Up	Neuronal death	miR‐204	50

Abbreviations: MALAT1, metastasis associated lung adenocarcinoma transcript 1; PI3K, phosphoinositide‐3‐kinase; PTEN, phosphatase and tensin homolog deleted on chromosome 10; XIST, X‐inactive specific transcript.

### 3.1 Glial activation

Glia could be activated within 1 day (microglial activation) and persist for months or even years (astrogliosis) after SCI.^38^, ^39^, ^40^, ^41^ As for different cellular subsets (eg M1 vs M2 phenotypes)^42^ and locations (eg scar vs spared tissue of lesions),^43^ glial cells exhibit distinct molecular properties and play different roles in inflammation, neuronal death and demyelination. A careful interpretation of cell‐type‐ or location‐dependent molecular functions will shed light on the pathologic mechanisms of SCI and provide potential therapeutic targets. In a rat model of acute contusive SCI, metastasis associated lung adenocarcinoma transcript 1 (MALAT1) was found to be significantly up‐regulated in contusion epicentre of spinal cord.^44^ Metastasis associated lung adenocarcinoma transcript 1 in turn ‘sponge’ miR‐199b and hence promote the production of pro‐inflammatory cytokines. Moreover, knockdown of spinal MALAT1 reduced the expression of Iba‐1 (microglial marker) and pro‐inflammatory cytokines in contusion epicentre, and improved locomotor function of hindlimb in the same model of SCI. However, the roles of MALAT1 in microglial polarization were not explored in this study. In another report,^45^ the expression of lncSCIR1 was found to be constantly down‐regulated on the 1st, 4th and 7th day after moderate contusive SCI. lncSCIR1 (long non‐coding spinal cord injury related 1) was inversely correlated to the expression of Bmp7 (bone morphogenetic protein 7) and Adm (Adrenomedullin), both of which have been reported to promote astrogliosis in the spinal cord,^46^, ^47^ indicating that lncSCIR1 might have a role in regulating astrocytes. Indeed, knockdown of lncSCIR1 was sufficient to promote the migration and proliferation of cultured astrocytes.^45^ However, most of the functional studies were performed in vitro. The distribution of lncSCIR1, ie scar or perilesional area, was unclear. The evidence that whether lncSCIR1 replenishment was beneficial for SCI was also lacking. Nonetheless, these studies provided preliminary evidence that lncRNAs might participate in gliogenesis after SCI.

### 3.2 Neuronal apoptosis

Neuronal death was probably the most evident alteration in SCI, especially in the acute phase. Modulators of neuronal apoptosis therefore drew continuous attentions from researchers. Through the re‐analysis of GEO dataset (accession GSE5296), XIST was identified as one of the up‐regulated lncRNAs with the highest fold‐changes in a mouse model of contusive SCI.^48^ Moreover, XIST knockdown exerted considerable neuroprotection via activating anti‐apoptotic phosphoinositide‐3‐kinase protein kinase B (PI3K)/AKT pathway in the injured spinal cord. Mechanistically, XIST knockdown led to the elevation of miR‐494, which then inhibited the phosphatase and tensin homolog deleted on chromosome 10 (PTEN) levels. The reduction of PTEN in turn activated the PI3K/AKT pathway and protected against neuronal apoptosis in the spinal cord.

Spinal cord ischaemic/reperfusion injury (SCIRI) is another common SCI model. Compared to sham operation, lncRNA CasC7 level was significantly decreased in the SCIRI group.^49^ Interestingly, hydrogen sulphide preconditioning, which could protect neurons from apoptotic cell death and reduce injury‐induced spinal cord infarction, reversed CasC7 expression alteration. CasC7 knockdown promoted miRNA‐30c expression and abrogated hydrogen sulphide‐mediated neuroprotection, suggesting that CasC7 was a novel modulator of neurodegeneration in SCI. Similarly, spinal MALAT1 was shown to be reduced in the model of SCIRI.^50^ In neurons, MALAT1 knockdown is sufficient to induce miR‐204‐dependent apoptosis. By contrast, MALAT1 overexpression reduced apoptosis and improved motor function in vivo. These results indicated that, in addition to CasC7, MALAT1 might function as another neuroprotective lncRNA. It should be noted that this finding appears to be opposite to the investigation of MALAT1 in microglia, in which MALAT1 was detrimental to neurons by driving microglia‐mediated inflammatory responses. This may be attributed to different models of injury (SCIRI vs contusive SCI).

Although several interesting findings have been presented, studies of lncRNAs molecular functions are still limited. First of all, most of the current studies appear to need more elaborate evidence and experimental designs. For instance, it was reported that different subsets of macrophages, ie M1 and M2 subsets, exerted neurotoxicity and regeneration actions to injured spinal cord respectively.^51^ None of the current studies had explored the lncRNAs distribution in different cell subsets, nor were investigating the potential roles of lncRNAs in distinct cellular functions. Secondly, only ceRNA‐like functions of lncRNAs have been explored by these studies. The conclusions were also made merely based on the findings at the molecular level. Functional assessments at the synaptic and behavioural levels are therefore necessary to generate convincing conclusions. In addition to ceRNA‐related function, the potential roles of these lncRNAs in the transcriptional modulation in SCI are also worthy of future investigations. Thirdly, although many pathological alterations, such as haemorrhage,^52^ demyelination^29^ and scar formation^53^ attract great attentions to researchers and clinicians, no studies have examined the roles of lncRNAs in these processes. Meanwhile, given that a large number of lncRNAs are involved in stem cell differentiation or neurogenesis,^54^ lncRNAs alone or as modulators of stem cell transplantation could be explored for SCI rehabilitation.

## 4 CONCLUSION

Spinal cord injury is still a tough clinical issue that needs intensive research. Previous studies have gradually disclosed the pathological changes of SCI and their underlying mechanism. Current investigations on the molecular properties of SCI are focusing on protein‐coding genes, yet the clinical translation is still not satisfactory. lncRNAs, via interacting with the coding gene network, participate in various cellular and tissue alterations in all stages of SCI. lncRNA deregulations therefore represent a new dimension of molecular mechanisms of SCI.

## CONFLICT OF INTEREST

There is no conflict of interest**.**

## AUTHORS CONTRIBUTION

Zheng Li, Idy HT Ho, Xingye Li, Derong Xu, William K.K. Wu, Matthew T.V. Chan, Shugang Li and Xiaodong Liu have all contributed to write, designed studies, analyzed results and write paper.

## DATA AVAILABILITY STATEMENT

I confirm that I have included a citation for available data in my references section.
